# Generation of Monoclonal Antibodies against Ag85A Antigen of *Mycobacterium tuberculosis* and Application in a Competitive ELISA for Serodiagnosis of Bovine Tuberculosis

**DOI:** 10.3389/fvets.2017.00107

**Published:** 2017-06-30

**Authors:** Zhengzhong Xu, Ting Hu, Aihong Xia, Xin Li, Ze Liu, Jingjing Min, Jingjing He, Chuang Meng, Yuelan Yin, Xiang Chen, Xinan Jiao

**Affiliations:** ^1^Jiangsu Key Laboratory of Zoonosis, Yangzhou University, Yangzhou, China; ^2^Key Laboratory of Prevention and Control of Biological Hazard Factors (Animal Origin) for Agrifood Safety and Quality, MOA, Yangzhou University, Yangzhou, China; ^3^Jiangsu Co-Innovation Center for Prevention and Control of Important Animal Infectious Diseases and Zoonoses, Yangzhou University, Yangzhou, China

**Keywords:** Mycobacterium tuberculosis, Ag85A, *Monoclonal antibody*, cross-react, competitive ELISA, bovine tuberculosis

## Abstract

The Ag85 complex functions as the main secretory protein of *Mycobacterium tuberculosis* (*M. tuberculosis*) and BCG. This complex is composed of the proteins, Ag85A, Ag85B, and Ag85C, with Ag85A thought to play the largest role within the complex. However, the lack of commercially available monoclonal antibodies (mAbs) against Ag85A still hinders the biological and applicative research on this protein. In this study, we developed and identified anti-Ag85A mAbs, and five hybridoma cells were established. Using the indirect immunofluorescence test, we found that two anti-Ag85A mAbs did not cross-react with Ag85B and/or Ag85C. In addition, we showed that all of the mAbs tested in this study are able to react with endogenous Ag85A protein in BCG and rBCG:Ag85A using indirect ELISA and Western blot analyses. A competitive ELISA (cELISA) based on mAb 3B8 was developed, the analyses of clinic serum samples from cattle with bovine tuberculosis (TB) and healthy cattle demonstrated that the sensitivity of the cELISA was 54.2% (26/48) and the specificity was 83.5% (167/200). This study demonstrated that the mAbs against Ag85A will provide useful reagents for further investigation into the function of the Ag85 complex and can be used for serodiagnosis of bovine TB.

## Introduction

Tuberculosis (TB) is an infectious disease that is widely prevalent throughout the entire world. Approximately one-third of the population of the world is infected with *Mycobacterium tuberculosis* (*M. tuberculosis*). In 2015, there were an estimated 10.4 million new cases of active TB across the world, and it was responsible for an estimated 1.4 million deaths globally in the same year (1–3).

The majority of secreted proteins found in *M. tuberculosis* culture filtrate have been shown to be generated by the Ag85 complex, a complex comprised three proteins from a 30- to 32-kDa protein family (Ag85A, Ag85B, and Ag85C). These three proteins are secreted into the culture medium in a 2:3:1 ratio (4, 5). The proteins of the Ag85 complex are encoded by three paralogous genes, *fbpA, fbpB*, and *fbpC*, all of which have been shown to be localized to distinct regions of the bacterial genome. *M. tuberculosis* Ag85A, Ag85B, and Ag85C are highly homologous on the DNA and amino acid level, with approximately 77% of amino acids shared between Ag85A and Ag85B, and about 71% amino acids shared between Ag85A and Ag85C (6).

The proteins comprising the Ag85 complex have been shown to be abundantly secreted in *M. tuberculosis*. These proteins play a key role in the final step of cell wall assembly and the maintenance of the bacterial cell envelope integrity by catalyzing the transfer of mycolic acid to the cell wall component, arabinogalactan. In addition, this complex has been shown to play an important role in the synthesis of trehalose dimycolate (7–9). Numerous studies to date have focused on the potential of Ag85 complex in vaccine development, diagnostics, and as a therapeutic drug target (10). Because the Ag85 complex has been shown to play a role in the catalysis of the biosynthesis of abundant cell envelope components, including TMM and TDM, there is great interest in Ag85 as a novel target for drug development (11). The *M. tuberculosis* Ag85 complex has been demonstrated to stimulate a strong humoral- and cell-mediated immune response (12, 13). Thus, Ag85A is considered to be one of the most popular TB vaccine candidates (14–16). In addition, the abundance of serum antibodies generated against the Ag85 complex in active TB patients provides further support that the Ag85 complex could also function as a promising diagnostic marker (5, 17).

There were already some works describing the production of monoclonal antibodies (mAbs) to Ag85 complex, nine mAbs were produced against *M. tuberculosis* Ag85 complex using isoelectric focusing combined with Western blot analysis, the results showed that one antibody was found to be specifically directed only against Ag85B (18). A method to select antibodies against any Ag85 complex using a novel combination of phage and yeast display was described (19). And antibodies to Ag85B of *M. tuberculosis* were produced and subsequently used to develop ELISA technique for detecting Ag85 in the culture filtrate (20). Up until this point, there have been no commercial specific mAbs available against Ag85A. The widely used anti-Ag85 mAb HYT 27 reacts strongly with *M. tuberculosis* Ag85C and weaker with Ag85A and Ag85B (21, 22). The rabbit polyclonal antibody against *M. tuberculosis* Ag85B is only specific for the Ag85B protein (23, 24). Thus, it is necessary to first develop a specific mAb against Ag85A to be used in both basic biological research and Ag85A applicative research.

In this study, we developed mAbs against recombinant Ag85A protein. We showed that all of the generated mAbs exhibit good reactivity with both recombinant Ag85A and endogenous Ag85A *via* indirect ELISA and Western blot techniques. And mAbs 1C6 and 3B8 were specific only for Ag85A, mAbs 2E6 and 2F2 cross-reacted with Ag85B or Ag85C, while mAb 3D9 react with Ag85A, Ag85B, and Ag85C. A competitive ELISA (cELISA) based on mAb 3B8 was developed, and the diagnostic specificity and sensitivity were 54.2% (26/48) and 83.5% (167/200), respectively. We anticipate that the mAbs generated against Ag85A will prove to be a valuable tool for the study of the biological function of the Ag85 complex. In addition, these antibodies hold great promise as tools that can be used toward the development of diagnostic methods and drug development for *M. tuberculosis*.

## Materials and Methods

### Construction of Recombinant Expression Vector

The *fbpA* gene was PCR amplified from chromosomal DNA isolated from the *M. tuberculosis* H37Rv strain. The sequences of the primer used for PCR amplification are as follows: sense primer, 5′-AAGCGGATCCATGTTTTCCCGGCCGGGCTTG-3′, antisense primer, 5′-AGTCGAATTCTGTTCGGAGCTAGGCGCCCTGGG-3′. Amplification reactions were carried out at 95°C for 5 min followed by 30 cycles at 94°C for 45 s, annealing at 55°C for 1 min, extension at 72°C for 2 min, and final extension at 72°C for 30 min. The generated gene fragments were then ligated to the T-cloning site of a pMD20-T vector (Takara, Japan). This was then isolated by digestion with *Bam*HI and *Eco*RI. The *fbpA* gene was then ligated into pET-30a and pGEX-6p-1 vectors to generate recombinant plasmids.

The genes *fbpA, fbpB*, and *fbpC* were fused genetically to GFP gene to generate GFP-*fbpA*, GFP-*fbpB*, and GFP-*fbpC* fragments, respectively, by the splice overlap extensioning PCR technique. Briefly, the GFP gene fragment was amplified from the EGFP plasmid by PCR using primers GFP-F1 and GFP-R1. The *fbpA, fbpB*, and *fbpC* genes were amplified from the genomic DNA of *M. tuberculosis* H37Rv strain by PCR, using primers *fbpA*-F2, *fbpA*-R2, *fbpB*-F2, *fbpB*-R2, *fbpC*-F2, and *fbpC*-R2. The sequence for the four pairs of primers was summarized in Table 1. After the first rounds of PCR using GFP-F1/GFP-R1, *fbpA*-F2/*fbpA*-R2, *fbpB*-F2/*fbpB*-R2, and *fbpC*-F2/*fbpC*-R2, the PCR products were gel purified. In a second round of PCR, using the resulting PCR products as templates, the GFP-*fbpA*, GFP-*fbpB*, and GFP-*fbpC* fusion genes were created by overlap PCR using primers GFP-F1/*fbpA*-R2, GFP-F1/*fbpB*-R2, and GFP-F1/*fbpC*-R2. The sequence GGGGSGGGGS was incorporated at the junction of the GFP fragment and *fbpA*/*fbpB*/*fbpC* gene as a flexible linker. The fusion genes were cloned into the pcDNA3.1(+) vector (Invitrogen, USA) between the *Eco*RI and *Xba* I restriction sites to generate pcDNA3.1-GFP-*fbpA*, pcDNA3.1-GFP-*fbpB*, and pcDNA3.1-GFP-*fbpC* constructs and confirmed by restriction endonuclease digestion and DNA sequencing.

**Table 1:** Primer sequences.

Primers	Sequences
GFP-F1	5′-TAGAATTCGCCACCATGGTGAGCAAGGGCGAGGAGCTG-3′
GFP-R1	5′-CACCGCCGCTTCCACCGCCACCCTTGTACAGCTCGTCCATGCCGAG-3′
*fbpA*-F2	5′-GTGGAAGCGGCGGTGGCGGAAGCATGCAGCTTGTTGACAGGGTTC-3′
*fbpA*-R2	5′-TATCTAGAGTTGTGTCTGTTCGGAGCTAGGC-3′
*fbpB*-F2	5′-GTGGAAGCGGCGGTGGCGGAAGCATGACAGACGTGAGCCGAAAGA-3′
*fbpB*-R2	5′-TATCTAGAAACCCTTCGGTTGATCCCGTCA-3′
*fbpC*-F2	5′-GTGGAAGCGGCGGTGGCGGAAGCATGACGTTCTTCGAACAGGT-3′
*fbpC*-R2	5′-TATCTAGAGATGCTGGCTTGCTGGCTCA-3′

### Expression and Purification of Recombinant Ag85A Protein

The pET-30a-*fbpA* and pGEX-6p-1-*fbpA* constructs were transformed into *Escherichia coli* strains BL21(DE3) and BL21, respectively. Transformed *E. coli* cells were cultured and recombinant protein expression was induced using 0.5 mM isopropyl-β-d-thiogalactopyranoside. The cells were then harvested and lysed by sonication on ice. The recombinant proteins, rHis-Ag85A and rGST-Ag85A, were purified from the lysate using the His-binding purification kit (Novagen, Germany) and GST-binding purification kit (GE, USA) according to the manufacturer’s instructions, respectively.

### Immunization of Mice and Establishment of Hybridomas

BALB/c mice (females, 6 weeks old) were injected subcutaneously with 80 µg of purified rGST-Ag85A protein mixed with Freund’s complete adjuvant (Sigma-Aldrich, USA) in a 1:1 volumetric ratio. A secondary immunization with the same antigen was also mixed with Freund’s incomplete adjuvant (Sigma-Aldrich, USA) in a 1:1 volumetric ratio, and it was given at 2-week intervals. One week following the secondary immunization, the serum titer was determined using an indirect ELISA method with purified rHis-Ag85A protein. The final booster immunization with the same antigen was injected intravenously 3 days prior to cell fusion, without any adjuvants. Finally, the splenocytes of BALB/c mice were collected and fused with SP2/0 myeloma cells using standard hybridoma methods (25). The mice were housed, handled, and immunized at our animal biosafety facilities, and all procedures were approved by the Institutional Animal Experimental Committee of Yangzhou University. All experiments were performed according to the national guidelines for animal welfare.

### Screening of Hybridoma Cells

To screen for positive hybridomas, titers of the hybridoma supernatants were determined using an indirect ELISA method with purified rHis-Ag85A protein. In these experiments, a polyclonal antibody against Ag85A protein was used as positive control, and the supernatant from SP2/0 myeloma cells was used as negative control. After subcloning cells two to three times, hybridoma cells that stably secreted antibody were established. Hybridoma cells were then intraperitoneally injected into BALB/c mice to induce the generation of ascites containing mAbs against Ag85A protein.

### Isotype and Titer Analysis

Both the class and subclass of the mAbs against Ag85A produced were determined using a mouse monoclonal antibody isotyping kit (Sigma-Aldrich, USA). Protocols were carried out according to the manufacturer’s instructions.

ELISA plates (Nunc, Denmark) were coated with purified rHis-Ag85A protein (1 µg/ml) and left overnight at 4°C. The next morning, plates were washed three times with PBST and blocked with phosphate-buffered saline (PBS) containing 2% bovine serum albumin (BSA) at 37°C for 2 h. Following the blocking step, the plates were washed three times with PBST. Plates were then incubated with serially diluted culture supernatant and ascites. Plates were incubated with primary antibody for 1 h at 37°C. HRP-conjugated goat anti-mouse IgG antibody (1:8,000 dilution) (Sigma-Aldrich, USA) was then added to each well (100 µl/well) and incubated for 1 h at 37°C. Finally, the TMB substrate was then added to the plates and incubated for 10 min, after which the plates were read at 450 nm.

### Western Blot Analysis

Purified rHis-Ag85A, rGST-Ag85A, wtBCG, and rBCG:Ag85A (26) were separated on 12% SDS-PAGE gels. Protein was then transferred to PVDF membrane. The membrane was then blocked for 2 h at room temperature with 2% BSA in PBS. The membrane was then washed three times with PBS containing 0.05% Tween 20 (PBST). Following the washes, the membrane was incubated with anti-Ag85A mAbs (1:1,000) at room temperature for 1 h. The membrane was then washed again three times in PBST, followed by incubation with HRP-conjugated goat anti-mouse secondary antibody (1:2,000) (Sigma-Aldrich, USA). Finally, the membrane was developed using 3,3′-diaminobenzidine tetrahydrochloride (Sigma-Aldrich, USA) and visualized using X-ray film.

### Indirect ELISA Assay

ELISA plates (Nunc, Denmark) were coated with MPT63, RpfE, CFP10-ESAT6, and Ag85A protein (1 µg/ml), respectively, and left overnight at 4°C. The next morning, plates were washed three times with PBST and blocked with PBS containing 2% BSA at 37°C for 2 h. Following the blocking step, the plates were washed three times with PBST. Plates were then incubated with the anti-Ag85A mAbs (1:2,000 dilution). Plates were incubated with primary antibody for 1 h at 37°C. HRP-conjugated goat anti-mouse IgG antibody (1:8,000 dilution) (Sigma-Aldrich, USA) was then added to each well (100 μl/well) and incubated for 1 h at 37°C. Finally, the TMB substrate was then added to the plates and incubated for 10 min, after which the plates were read at 450 nm.

### Transient Transfection and Indirect Immunofluorescence Test

One day prior to transfection, HEK293T cells were plated in 24-well plates in complete DMEM at a density of 1 × 10^5^ cells/well. Cells were permitted to attach to the plate overnight at 37°C in a 5% CO_2_ atmosphere. The following day, HEK293T monolayers were transfected with the pcDNA3.1-GFP-*fbpA*, pcDNA3.1-GFP-*fbpB*, and pcDNA3.1-GFP-*fbpC* constructs using Lipofectamine reagent 3000 (Invitrogen, USA) according to the manufacturer’s instructions.

Twenty-four hours posttransfection, HEK293T cells were washed twice with PBS. Cells were then fixed with methanol for 15 min at room temperature. Following fixation, the cells were incubated with anti-Ag85A mAbs for 2 h at room temperature. The cells were then washed with PBS and incubated with Alexa Fluor^®^ 586 conjugated goat anti-mouse IgG secondary antibody (Life Technology, USA) for 1 h. Cell staining was observed using fluorescence microscopy.

### Detection of Ag85A Protein in BCG and rBCG:Ag85A

ELISA plates (Nunc, Denmark) were first treated with 5% glutaraldehyde (100 µl/well) and incubated at 37°C for 2 h. Plates were then washed three times with PBST. Following the washes, plates were coated with 1 × 10^6^ colony-forming units of BCG or rBCG:Ag85A bacteria (100 µl/well) and left overnight at 56°C. The plates were washed with PBST and blocked with PBS containing 2% BSA at 37°C for 1 h. Then, the plates were washed and incubated with the mAbs for 2 h at 37°C. HRP-conjugated goat anti-mouse IgG antibody (1:8,000 dilution) (Sigma-Aldrich, USA) was added to each well (100 µl/well) and incubated for 1 h at 37°C. Finally, the TMB substrate was then added to the plates and incubated for 10 min, after which the plates were read at 450 nm.

### Protocol of cELISA Assay

ELISA plates (Nunc, Denmark) were coated with rHis-Ag85A protein (1 µg/ml) overnight at 4°C. The next morning, plates were washed three times with PBST and blocked with PBS containing 2% BSA at 37°C for 2 h. Following the blocking step, the plates were washed three times with PBST. Test and control sera samples were diluted 1:100 in PBS and added to each well (50 µl/well), and HRP-labeled anti-Ag85A antibody 3B8 were diluted (1:1,000) and added to each well (50 µl/well), followed by incubation at 37°C for 2 h and washing. Finally, the TMB substrate was then added to the plates and incubated for 10 min, after which the plates were read at 450 nm. Results were calculated based on the OD450 values of the negative control serum sample (*N*) and test serum sample (*S*) using the following formula: % inhibition = [(*N* − *S*)/*N*] × 100%. A total of 200 healthy bovine serum samples were tested for determination of cutoff point. The mean inhibition rate + 3× SD was cutoff point (Cn). If the inhibition rate ≥Cn, the bovine TB antibody reaction is positive, if the inhibition rate <Cn, the bovine TB antibody reaction is negative.

### Evaluation of cELISA for Detection of Anti-Ag85A Antibody

Total of 298 serum samples were harvested from dairy farms, of which 76 samples are positive and 222 samples are negative identified by PPD skin test, 70 samples are positive and 228 samples are negative identified by interferon-gamma release assay (IFN-γ assay) (Prionics, Switzerland). Bovine serum samples were diluted 100-fold and the inhibition rate was measured using cELISA method based on the criteria described above to determine the sensitivity and specificity of the assay.

### Statistical Analysis

All data are expressed as mean ± SEM. Statistical analysis was performed using a Student’s *t*-test. A value of *P < 0.05, **P < 0.01, ***P < 0.001 was considered statistically significant.

## Results

### Isotype and Titer Analysis of mAbs

Following two to three rounds of cell subcloning and detection, hybridoma cells producing anti-Ag85A mAbs were established and named 1C6, 2E6, 2F2, 3B8, and 3D9. Isotype analysis of the mAbs produced against Ag85A protein was determined using a mouse mAb isotyping kit. These results demonstrated that all nine mAbs belong to the IgG1 isotype (Table 2). In addition, ELISA results suggested that both hybridoma supernatant and ascites possessed high titer levels (Table 2).

**Table 2:** Identification and characteristic of anti-Ag85A monoclonal antibodies (mAbs).

mAbs	Isotype	Supernatant titer	Ascites titer
1C6	IgG1	1:81,902	1:8,192,000
2E6	IgG1	1:10,240	1:16,384,000
2F2	IgG1	1:327,680	1:16,384,000
3B8	IgG1	1:20,480	1:1,024,000
3D9	IgG1	1:40,960	1:8,192,000

### Western Blot Analysis

**Figure 1:**
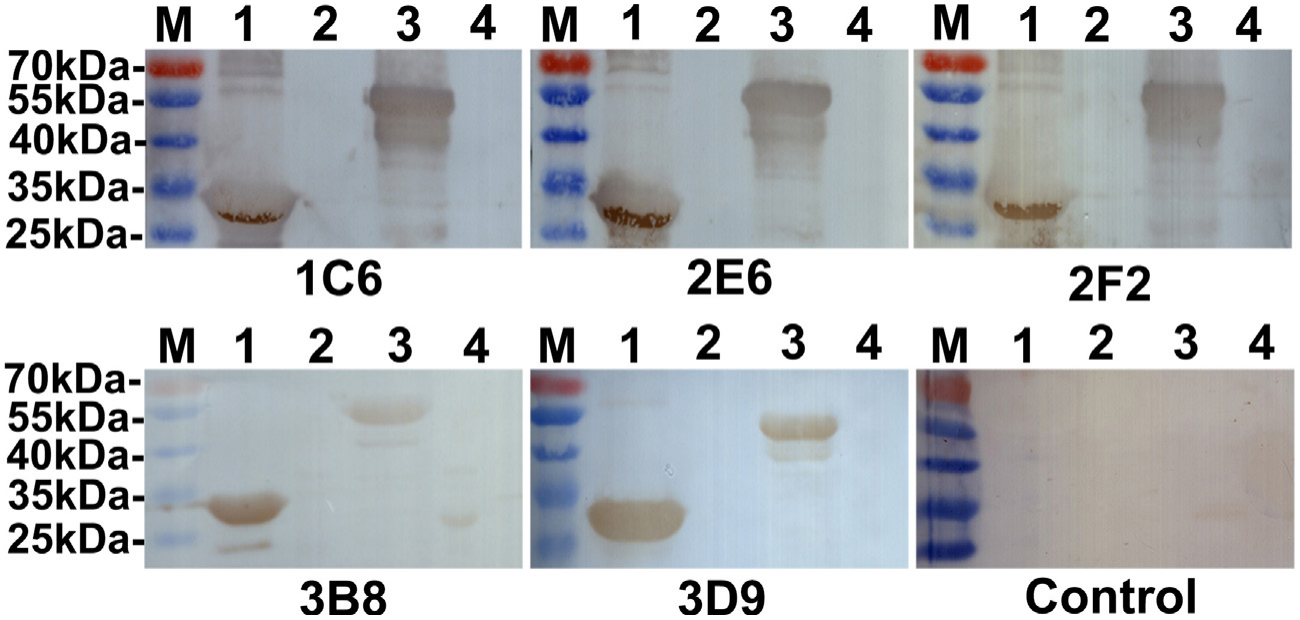
Western blot analysis. All anti-Ag85A monoclonal antibodies (mAbs) recognized rHis-Ag85A protein (lane 1) and rGST-Ag85A protein (lane 3). Anti-Ag85A mAbs did not react with BL21(DE3) (pET-30a) (lane 2) and BL21(pGEX-6p-1) (lane 4). The control group was recombinant protein react with SP2/0 ascites.

The reactivity of anti-Ag85A mAbs against recombinant Ag85A protein was studied using Western blot analysis. We show that all anti-Ag85A mAbs react with rHis-Ag85A and rGST-Ag85A protein (Figure 1), with all the mAbs exhibiting good reactivity.

### Indirect ELISA Assay

**Figure 2:**
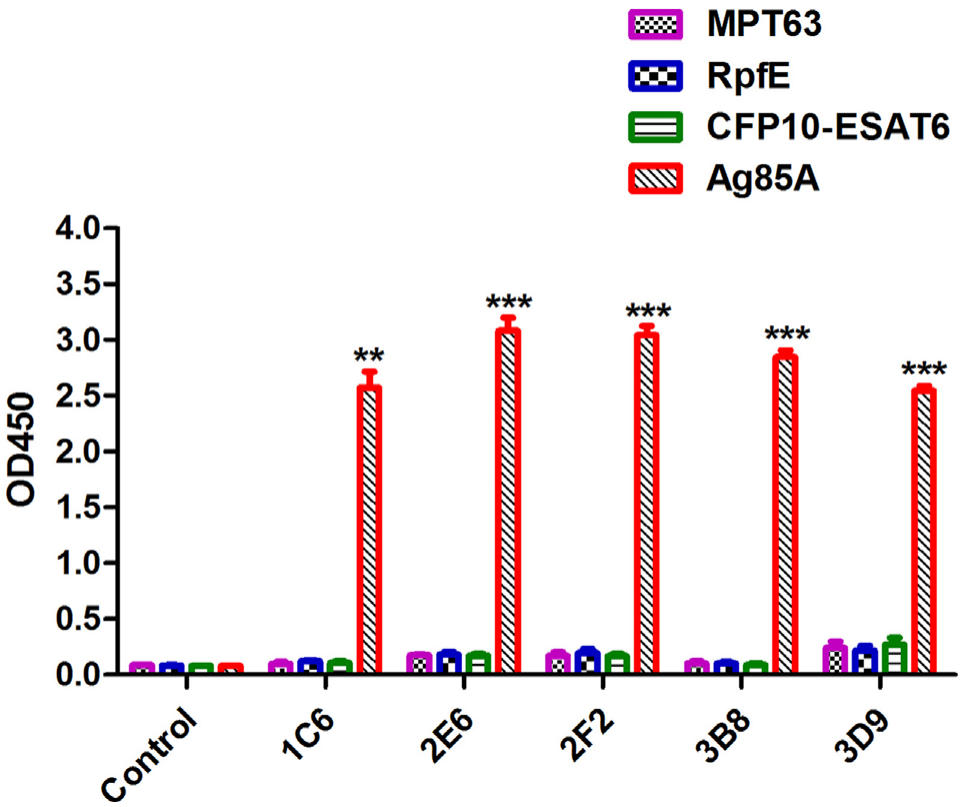
Indirect ELISA assay. The specificity of anti-Ag85A monoclonal antibodies (mAbs) was detected by indirect ELISA. All anti-Ag85A mAbs strongly recognized Ag85A protein, but did not react with MPT63, RpfE, and CFP10-ESAT6 protein. The control group was recombinant protein react with SP2/0 ascites. The control group was SP2/0 ascites control. Data depicted are the mean values ± SEM. Statistical significance was determined by a Student’s *t*-test (**P < 0.01, ***P < 0.001).

In order to evaluate the specificity of anti-Ag85A mAbs, the cross-reactivity of anti-Ag85A mAbs against other protein from mycobacteria was detected by indirect ELISA assay. The results showed that anti-Ag85A mAbs 1C6, 2E6, 2F2, 3B8, and 3D9 did not react with MTP63, RpfE, and CFP10-ESAT6 (Figure 2). All the mAbs did not react with selected mycobacteria protein, suggesting these mAbs are specific to Ag85A protein.

### Indirect Immunofluorescence Test

**Figure 3:**
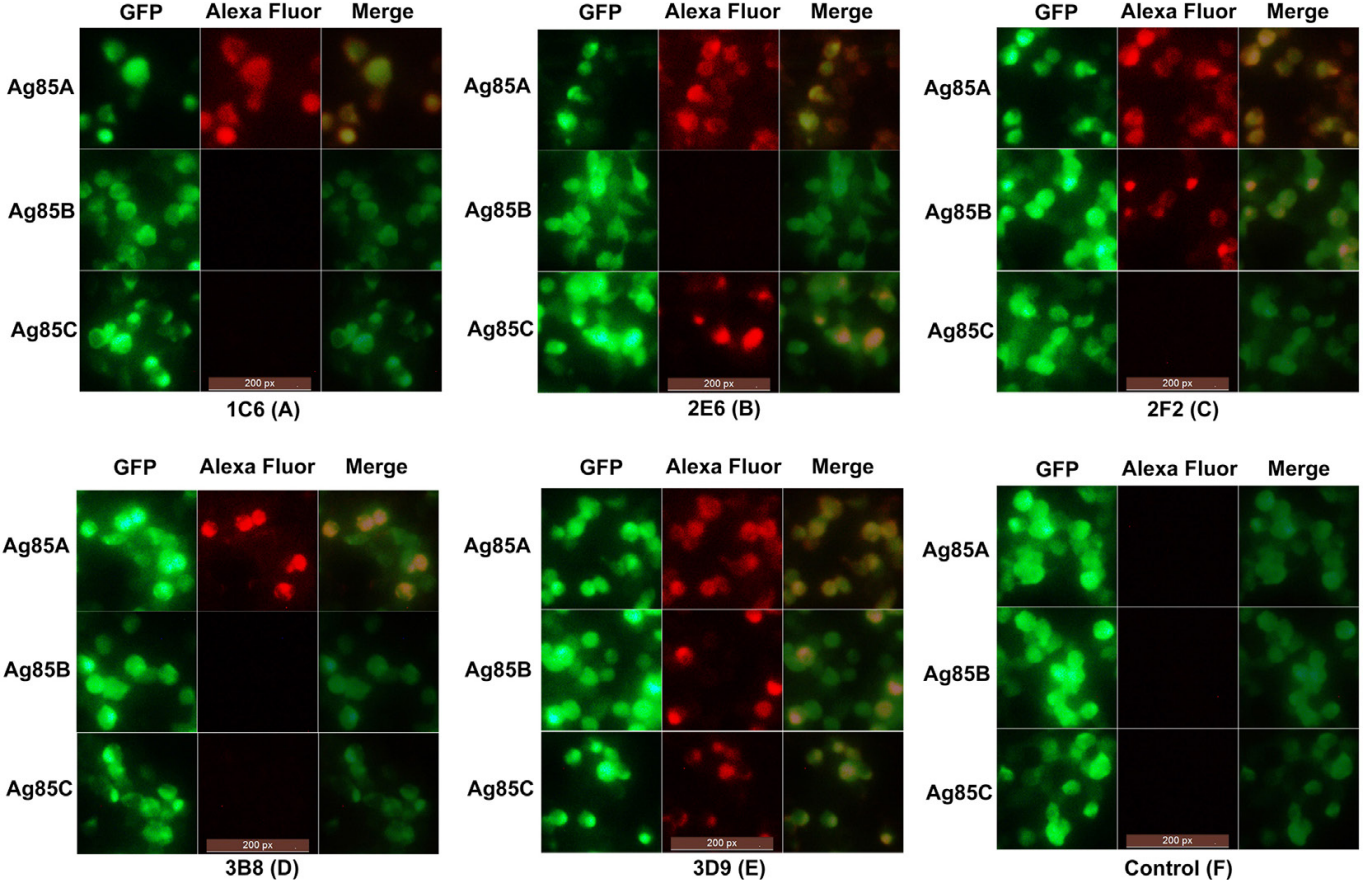
Indirect immunofluorescence test. HEK293T cells transfected with pcDNA3.1-*fbpA*-GFP, pcDNA3.1-*fbpB*-GFP, and pcDNA3.1-*fbpC*-GFP constructs were stained with anti-Ag85A monoclonal antibodies (mAbs) and Alexa Fluor 568-conjugated anti-mouse IgG antibody, respectively. Following staining with mAbs 1C6 and 3B8, only HEK293T cells transfected with pcDNA3.1-*fbpA*-GFP exhibited yellow fluorescence **(A,D)**. However, HEK293T cells transfected with pcDNA3.1-*fbpB*-GFP and/or pcDNA3.1-*fbpC*-GFP also exhibited yellow fluorescence following staining with mAbs 2E6, 2F2, and 3D9 **(B,C,E)**. The control group was HEK293T cells stained with SP2/0 ascites **(F)**.

In order to evaluate the cross-reactivity of anti-Ag85A mAbs with the Ag85 complex, HEK293T cells were transfected with pcDNA3.1-*fbpA*-GFP, pcDNA3.1-*fbpB*-GFP, and pcDNA3.1-*fbpC*-GFP constructs. Cells were then stained with the anti-Ag85A mAbs. We show that following staining with mAbs 1C6 and 3B8, only HEK293T cells transfected with pcDNA3.1-*fbpA*-GFP exhibited yellow fluorescence (Figures 3A,D). However, HEK293T cells transfected with pcDNA3.1-*fbpB*-GFP and/or pcDNA3.1-*fbpC*-GFP also exhibited yellow fluorescence following staining with the mAbs 2E6, 2F2, and 3D9 (Figures 3B,C,E). The HEK293T cells in the control group did not exhibited yellow fluorescence (Figure 3F). This suggests that a subset of anti-Ag85A mAbs cross-react with Ag85B and/or Ag85C. The mAbs 2F2 and 2E6 cross-react with Ag85B and Ag85C, respectively, and the mAbs 3D9 can react with Ag85A, Ag85B, and Ag85C.

### Detection of Ag85A Protein in BCG and rBCG:Ag85A

**Figure 4:**
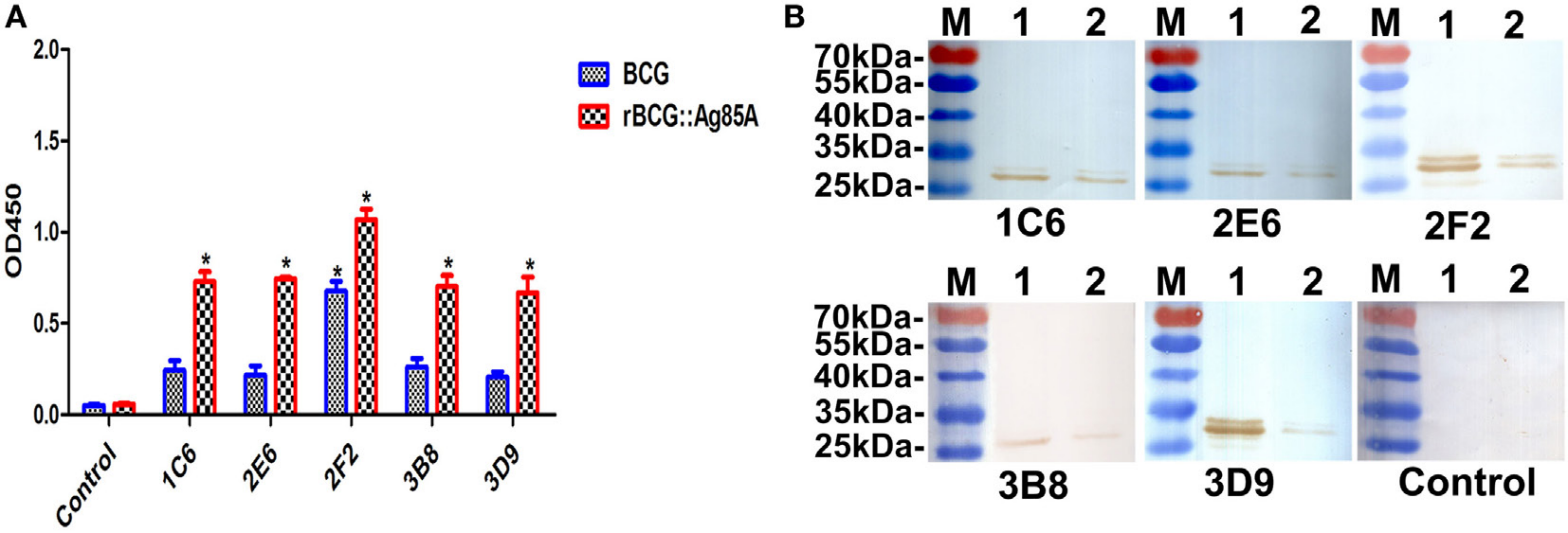
Detection of Ag85A protein in BCG and rBCG:Ag85A. BCG and rBCG:Ag85A were detected using indirect ELISA and Western blot. All anti-Ag85A monoclonal antibodies (mAbs) were able to react with endogenous Ag85A protein in BCG and rBCG:Ag85A group **(A)**. All of the mAbs were able to recognize endogenous Ag85A protein in rBCG:Ag85A [**(B)** lane 1] and BCG [**(B)** lane 2]. The control group was SP2/0 ascites control. Data depicted are the mean values ± SEM. Statistical significance was determined by a Student’s *t*-test (*P < 0.05).

To determine whether the generated mAbs can also combine with endogenous Ag85A protein in BCG, we used Western blot and indirect ELISA methods to detect BCG and rBCG:Ag85A. We showed that all anti-Ag85A mAbs reacted with endogenous Ag85A protein in BCG and rBCG:Ag85A, the 31-kDa Ag85A protein levels are increased twofold in rBCG:Ag85A compared with those of BCG (Figures 4A,B). We also note that these mAbs reacted with other protein bands, which could represent Ag85B and/or Ag85C protein (Figure 4B), respectively.

#### Development of cELISA for Detection of Anti-Ag85A Antibody

A cELISA for detection of anti-Ag85A antibody was established. The rHis-Ag85A protein was coated on the ELISA plate as capture antigen, and anti-Ag85A antibody clone 3B8 was used as a detection antibody. The OD_450_ value was detected for 200 healthy bovine serum samples, the mean inhibition rate was 0.162 with a SD of 0.029, the Cn value was 0.249 (≈25%). A total of 248 serum samples (48 bovine TB positive samples and 200 bovine TB negative samples detected by both skin test and IFN-γ assay) were detected, the inhibition rate of 26 samples were above 25%, and the inhibition rate of 167 samples were below 25%, the results indicated that the diagnostic specificity and sensitivity were 54.2% (26/48) and 83.5% (167/200), respectively. A total of 298 serum samples (76 positive by skin test; 222 negative by skin test) were analyzed, the cELISA results were compared with those of the skin test. 45 skin test positive serum samples were also positive by cELISA, with a positive coincidence of 54.2% (90/166). And 177 skin test negative serum samples were negative by cELISA, with a negative coincidence of 82.3% (354/430). Therefore, the total coincidence of the cELISA method and skin test was 74.5% (Table 3). And the results were also compared with the IFN-γ assay, 298 serum samples (70 positive by IFN-γ assay; 228 negative by IFN-γ assay) were detected, 38 serum samples were also positive by cELISA with a positive coincidence of 47.5% (76/160), and 176 serum samples were also negative by cELISA with a negative coincidence of 80.7% (352/436). Therefore, the total coincidence of the cELISA assay and IFN-γ assay was 71.8% (Table 4).

**Table 3:** Comparative result of competitive ELISA (cELISA) assay and skin test.

		cELISA assay		Total
		*P*	*N*	
Skin test	*P*	45	31	76
	*N*	45	177	222
Total		90	208	298

**Table 4:** Comparative result of competitive ELISA (cELISA) assay and IFN-γ assay.

		cELISA assay		Total
		*P*	*N*	
IFN-γ assay	*P*	38	32	70
	*N*	52	176	228
Total		90	208	298

## Discussion

The Ag85A protein is a member of the Ag85 complex, a 30- to 32-kDa family of three proteins (Ag85A, Ag85B, and Ag85C). All three members of the Ag85 complex have been shown to exhibit mycolyltransferase activity (27). Interestingly, Ag85A has also been shown to stimulate production of Th1 cytokines and CTL activity. Because this protein generates a strong immune response, it is thought to be an ideal candidate antigen for the development of novel vaccines (28, 29).

However, there were still no commercial mAbs available against Ag85A. Thus, it may hinder the biological research and applicative research of Ag85A. The widely used commercial anti-Ag85 antibody HYT 27 only reacts strongly with *M. tuberculosis* Ag85C protein. The rabbit polyclonal antibody against *M. tuberculosis* Ag85B is only specific for the Ag85B protein.

Here, we used rGST-Ag85A protein as an immune antigen and rHis-Ag85A protein as a detective antigen. A total of five mAbs were established, and named as 1C6, 2E6, 2F2, 3B8, and 3D9. All nine mAbs used in this study were determined to be of the IgG1 isotype. Analysis of the generated mAbs showed that both hybridoma supernatant and ascites had a high titer. In addition, we show through Western blot analysis that all of the mAbs generated in this study are able to react with recombinant Ag85A protein. All the mAbs did not react with selected mycobacteria protein, suggesting these mAbs are specific to Ag85A protein.

*Mycobacterium tuberculosis* Ag85A, Ag85B, and Ag85C are highly homologous on the DNA and amino acid level (6). In order to evaluate the cross-reactivity of anti-Ag85A mAbs with the Ag85 complex, the HEK293T cells transfected with pcDNA3.1-*fbpA*-GFP, pcDNA3.1-*fbpB*-GFP, and pcDNA3.1-*fbpC*-GFP constructs were detected by indirect immunofluorescence, respectively. The mAbs 1C6 and 3B8 were specific only for Ag85A, the mAbs 2F2, 2E6, and 3D9 cross-react with Ag85B and/or Ag85C, respectively. The results suggested that a subset of the anti-Ag85A mAbs is able to cross-react with Ag85B and/or Ag85C. It is likely that some mAbs could recognize the same epitopes of Ag85A, Ag85B, and Ag85C protein. It may be beneficial to the wide employment of anti-Ag85A mAbs.

In previous works, we developed many mAbs using recombinant protein expressed in *E. coli*. All of these mAbs may have good reactivity against recombinant proteins but may not be reactive against the natural proteins (30). As a result, we identified the reactivity of anti-Ag85A mAbs against BCG. The indirect ELISA and Western blot analysis showed that all of the generated mAbs are able to react with endogenous Ag85A protein in BCG and rBCG:Ag85A. Currently, several serological tests with promising accuracy have recently emerged, many ELISA technology has been intensively established for the detection of serum antibodies of bovine TB (31–34). In this research, a cELISA based on mAb 3B8 was developed, the analyses of clinic serum samples from cattles with bovine TB and healthy cattles demonstrated that the diagnostic specificity and sensitivity were 54.2% (26/48) and 83.5% (167/200), respectively, and the total coincidence of the cELISA method with skin test and IFN-γ assay was 74.5 and 71.8%, respectively. Although the poor sensitivity of antibody-based ELISA methods has prevented widespread use of these assays for early detection of bovine TB, such as the sensitivity of an iELISA established with rM70-83-E6 as the diagnostic antigen was 69.4% (59/85) and the specificity was 96.0% (96/100) (32), an ELISA technology that detects antibody to *M. bovis* antigens MPB83 and MPB70 was established, and the sensitivity and specificity of the ELISA assay with naturally infected cattle were 63 and 98%, respectively (34). However, antibody responses to *M. bovis* well correlate with *M. bovis*-elicited pathology and *M. bovis* antigen burden (35), antibody response-based assays may be used in a wide range of applications and a supplemental test to cell-mediated response-based assays.

Overall, the mAbs generated against Ag85A will prove to be a valuable tool for the study of the subcellular localization and biological function of the Ag85 complex; in addition, these antibodies could act as important materials for the development of a diagnostic method for TB, as well as identification of novel drug candidates for the treatment of this infectious disease.

## Author Contributions

ZX, XC, and XJ designed the experiments. ZX, TH, AX, XL, ZL, JM, JH, and CM performed the experiments and analyzed the data. YY, XC, and XJ contributed reagents/materials/analysis tools. ZX, XC, and XJ wrote and revised the paper.

## Conflict of Interest Statement

The authors declare that the research was conducted in the absence of any commercial or financial relationships that could be construed as a potential conflict of interest.

## References

[1] WHO. Global tuberculosis report 2016. World Health Organisation Report. (2016). Available from: http://www.who.int/tb/publications/global_report/en/

[2] Yates TA Khan PY Knight GM Taylor JG McHugh TD Lipman M The transmission of *Mycobacterium tuberculosis* in high burden settings. Lancet Infect Dis (2016) 16:227–38. 10.1016/S1473-3099(15)00499-5 26867464

[3] Rappuoli R Changing route: aerosol vaccine against tuberculosis. Lancet Infect Dis (2014) 14:901–2. 10.1016/S1473-3099(14)70886-2 25151224

[4] Wiker HG Harboe M. The antigen 85 complex: a major secretion product of *Mycobacterium tuberculosis*. Microbiol Rev (1992) 56:648–61. 1480113 10.1128/mr.56.4.648-661.1992 PMC372892

[5] Bekmurzayeva A Sypabekova M Kanayeva D Tuberculosis diagnosis using immunodominant, secreted antigens of *Mycobacterium tuberculosis*. Tuberculosis (Edinb) (2013) 93:381–8. 10.1016/j.tube.2013.03.003 23602700

[6] Content J de la Cuvellerie A De Wit L Vincent-Levy-Frebault V Ooms J De Bruyn J. The genes coding for the antigen 85 complexes of *Mycobacterium tuberculosis* and *Mycobacterium bovis* BCG are members of a gene family: cloning, sequence determination, and genomic organization of the gene coding for antigen 85-C of *M. tuberculosis*. Infect Immun (1991) 59:3205–12. 1715324 10.1128/iai.59.9.3205-3212.1991 PMC258154

[7] Belisle JT Vissa VD Sievert T Takayama K Brennan PJ Besra GS. Role of the major antigen of *Mycobacterium tuberculosis* in cell wall biogenesis. Science (1997) 276:1420–2. 10.1126/science.276.5317.1420 9162010

[8] Swierzko AS Bartlomiejczyk MA Brzostek A Lukasiewicz J Michalski M Dziadek J Mycobacterial antigen 85 complex (Ag85) as a target for ficolins and mannose-binding lectin. Int J Med Microbiol (2016) 306:212–21. 10.1016/j.ijmm.2016.04.004 27141819

[9] Elamin AA Stehr M Spallek R Rohde M Singh M. The *Mycobacterium tuberculosis* Ag85A is a novel diacylglycerol acyltransferase involved in lipid body formation. Mol Microbiol (2011) 81:1577–92. 10.1111/j.1365-2958.2011.07792.x 21819455

[10] Tang X Deng W Xie J. Novel insights into *Mycobacterium* antigen Ag85 biology and implications in countermeasures for *M. tuberculosis*. Crit Rev Eukaryot Gene Expr (2012) 22:179–87. 10.1615/CritRevEukarGeneExpr.v22.i3.10 23140159

[11] Elamin AA Stehr M Oehlmann W Singh M. The mycolyltransferase 85A, a putative drug target of *Mycobacterium tuberculosis*: development of a novel assay and quantification of glycolipid-status of the mycobacterial cell wall. J Microbiol Methods (2009) 79:358–63. 10.1016/j.mimet.2009.10.010 19857528

[12] Rosseels V Marche S Roupie V Govaerts M Godfroid J Walravens K Members of the 30- to 32-kilodalton mycolyl transferase family (Ag85) from culture filtrate of *Mycobacterium avium* subsp. paratuberculosis are immunodominant Th1-type antigens recognized early upon infection in mice and cattle. Infect Immun (2006) 74:202–12. 10.1128/IAI.74.1.202-212.2006 16368974 PMC1346609

[13] Macedo GC Bozzi A Weinreich HR Bafica A Teixeira HC Oliveira SC Human T cell and antibody-mediated responses to the *Mycobacterium tuberculosis* recombinant 85A, 85B, and ESAT-6 antigens. Clin Dev Immunol (2011) 2011:351573 10.1155/2011/351573 21253450 PMC3023041

[14] Metcalfe HJ Steinbach S Jones GJ Connelley T Morrison WT Vordermeier M Protection associated with a TB vaccine is linked to increased frequency of Ag85A-specific CD4(+) T cells but no increase in avidity for Ag85A. Vaccine (2016) 34:4520–25. 10.1016/j.vaccine.2016.07.055 27498622 PMC5009893

[15] Harris SA Meyer J Satti I Marsay L Poulton ID Tanner R Evaluation of a human BCG challenge model to assess antimycobacterial immunity induced by BCG and a candidate tuberculosis vaccine, MVA85A, alone and in combination. J Infect Dis (2014) 209:1259–68. 10.1093/infdis/jit647 24273174 PMC3969545

[16] Jeyanathan M Damjanovic D Yao Y Bramson J Smaill F Xing Z. Induction of an immune-protective T-cell repertoire with diverse genetic coverage by a novel viral-vectored tuberculosis vaccine in humans. J Infect Dis (2016) 214:1996–2005. 10.1093/infdis/jiw467 27703038 PMC5142089

[17] Kashyap RS Rajan AN Ramteke SS Agrawal VS Kelkar SS Purohit HJ Diagnosis of tuberculosis in an Indian population by an indirect ELISA protocol based on detection of antigen 85 complex: a prospective cohort study. BMC Infect Dis (2007) 7:74 10.1186/1471-2334-7-74 17620147 PMC1933431

[18] Drowart A De Bruyn J Huygen K Damiani G Godfrey HP Stelandre M Isoelectrophoretic characterization of protein antigens present in mycobacterial culture filtrates and recognized by monoclonal antibodies directed against the *Mycobacterium bovis* BCG antigen 85 complex. Scand J Immunol (1992) 36:697–702. 10.1111/j.1365-3083.1992.tb03130.x 1439581

[19] Ferrara F Naranjo LA Kumar S Gaiotto T Mukundan H Swanson B Using phage and yeast display to select hundreds of monoclonal antibodies: application to antigen 85, a tuberculosis biomarker. PLoS One (2012) 7:e49535. 10.1371/journal.pone.0049535 23166701 PMC3498134

[20] Phunpae P Chanwong S Tayapiwatana C Apiratmateekul N Makeudom A Kasinrerk W. Rapid diagnosis of tuberculosis by identification of antigen 85 in mycobacterial culture system. Diagn Microbiol Infect Dis (2014) 78:242–8. 10.1016/j.diagmicrobio.2013.11.028 24418370

[21] Solans L Gonzalo-Asensio J Sala C Benjak A Uplekar S Rougemont J The PhoP-dependent ncRNA Mcr7 modulates the TAT secretion system in *Mycobacterium tuberculosis*. PLoS Pathog (2014) 10:e1004183. 10.1371/journal.ppat.1004183 24874799 PMC4038636

[22] Rengarajan J Murphy E Park A Krone CL Hett EC Bloom BR *Mycobacterium tuberculosis* Rv2224c modulates innate immune responses. Proc Natl Acad Sci U S A (2008) 105:264–9. 10.1073/pnas.0710601105 18172199 PMC2224198

[23] Torabi-Parizi P Vrisekoop N Kastenmuller W Gerner MY Egen JG Germain RN. Pathogen-related differences in the abundance of presented antigen are reflected in CD4+ T cell dynamic behavior and effector function in the lung. J Immunol (2014) 192:1651–60. 10.4049/jimmunol.1301743 24431231 PMC3923305

[24] Rath P Huang C Wang T Wang T Li H Prados-Rosales R Genetic regulation of vesiculogenesis and immunomodulation in *Mycobacterium tuberculosis*. Proc Natl Acad Sci U S A (2013) 110:E4790–7. 10.1073/pnas.1320118110 24248369 PMC3856836

[25] Chen X Ou Z Xie XL Xu ZZ Jiao XA. Preparation of monoclonal antibodies against *Mycobacterium tuberculosis* TB10.4 antigen. Monoclon Antib Immunodiagn Immunother (2014) 33:444–7. 10.1089/mab.2014.0039 25545212

[26] Xu ZZ Chen X Hu T Meng C Wang XB Rao Y Evaluation of immunogenicity and protective efficacy elicited by *Mycobacterium bovis* BCG overexpressing Ag85A protein against *Mycobacterium tuberculosis* aerosol infection. Front Cell Infect Microbiol (2016) 6:3. 10.3389/fcimb.2016.00003 26858942 PMC4729882

[27] Daffe M The mycobacterial antigens 85 complex – from structure to function and beyond. Trends Microbiol (2000) 8:438–40. 10.1016/S0966-842X(00)01844-8 11044671

[28] Denis O Tanghe A Palfliet K Jurion F van den Berg TP Vanonckelen A Vaccination with plasmid DNA encoding mycobacterial antigen 85A stimulates a CD4+ and CD8+ T-cell epitopic repertoire broader than that stimulated by *Mycobacterium tuberculosis* H37Rv infection. Infect Immun (1998) 66:1527–33. 9529077 10.1128/iai.66.4.1527-1533.1998 PMC108084

[29] Kamath AT Rochat AF Valenti MP Agger EM Lingnau K Andersen P Adult-like anti-mycobacterial T cell and in vivo dendritic cell responses following neonatal immunization with Ag85B-ESAT-6 in the IC31 adjuvant. PLoS One (2008) 3:e3683. 10.1371/journal.pone.0003683 18997860 PMC2577009

[30] Xu Z Shan F Shan F Meng C Zhou X Zhang X Generation and application of a 293 cell line stably expressing bovine interferon-gamma. Protein Expr Purif (2014) 99:131–7. 10.1016/j.pep.2014.04.012 24794970

[31] Casal C Infantes JA Risalde MA Diez-Guerrier A Dominguez M Moreno I Antibody detection tests improve the sensitivity of tuberculosis diagnosis in cattle. Res Vet Sci (2017) 112:214–21. 10.1016/j.rvsc.2017.05.012 28521256

[32] Liu S Guo S Wang C Shao M Zhang X Guo Y A novel fusion protein-based indirect enzyme-linked immunosorbent assay for the detection of bovine tuberculosis. Tuberculosis (Edinb) (2007) 87:212–7. 10.1016/j.tube.2006.07.007 17023217

[33] Green LR Jones CC Sherwood AL Garkavi IV Cangelosi GA Thacker TC Single-antigen serological testing for bovine tuberculosis. Clin Vaccine Immunol (2009) 16:1309–13. 10.1128/CVI.00028-09 19605596 PMC2745019

[34] Waters WR Buddle BM Vordermeier HM Gormley E Palmer MV Thacker TC Development and evaluation of an enzyme-linked immunosorbent assay for use in the detection of bovine tuberculosis in cattle. Clin Vaccine Immunol (2011) 18:1882–8. 10.1128/CVI.05343-11 21918115 PMC3209037

[35] Waters WR Whelan AO Lyashchenko KP Greenwald R Palmer MV Harris BN Immune responses in cattle inoculated with *Mycobacterium bovis, Mycobacterium tuberculosis*, or *Mycobacterium kansasii*. Clin Vaccine Immunol (2010) 17:247–52. 10.1128/CVI.00442-09 20007361 PMC2815522

